# Influence of the Use of Transepithelial Abutments vs. Titanium Base Abutments on Microgap Formation at the Dental Implant–Abutment Interface: An In Vitro Study

**DOI:** 10.3390/ma16196532

**Published:** 2023-10-01

**Authors:** Rocío Cascos, Alicia Celemín-Viñuela, Nataly Mory-Rubiños, Cristina Gómez-Polo, Rocío Ortega, Rubén Agustín-Panadero, Miguel Gómez-Polo

**Affiliations:** 1 Department of Conservative Dentistry and Orofacial Prosthodontics, Faculty of Dentistry, Complutense University of Madrid, 28040 Madrid, Spain; acelemin@ucm.es (A.C.-V.); natmory@ucm.es (N.M.-R.); mgomezpo@ucm.es (M.G.-P.); 2 Department of Nursing and Estomatology, Faculty of Health Sciences, Rey Juan Carlos University, 28922 Madrid, Spain; 3 Department of Prosthetic Dentistry, School of Dentistry, European University of Madrid, 28670 Madrid, Spain; rocio.ortega@universidadeuropea.es; 4 Department of Surgery, Faculty of Medicine, University of Salamanca, 37007 Salamanca, Spain; crisgodent@usal.es; 5 Prosthodontic and Occlusion Unit, Department of Stomatology, Faculty of Medicine and Dentistry, Universitat de València, 46010 Valencia, Spain; ruben.agustin@uv.es

**Keywords:** dental implants, dental abutments, dental implant–abutment design, dental prosthesis, implant-supported, scanning electron microscopy

## Abstract

This in vitro study aimed to assess the presence of microgaps at the implant–abutment interface in monolithic zirconia partial implant-supported fixed prostheses on transepithelial abutments versus Ti-base abutments. Methods: Sixty conical connection dental implants were divided into two groups (*n* = 30). The control group consisted of three-unit bridge monolithic zirconia connected to two implants by a transepithelial abutment. The test group consisted of monolithic zirconia three-unit restoration connected to two implants directly by a titanium base (Ti-base) abutment. The sample was subjected to thermocycling (10,000 cycles at 5 °C to 55 °C, dwelling time 50 s) and chewing simulation (300,000 cycles, under 200 N at frequencies of 2 Hz, at a 30° angle). The microgap was evaluated at six points (mesiobuccal, buccal, distobuccal, mesiolingual, lingual, and distolingual) of each implant–abutment interface by using a scanning electron microscope (SEM). The data were analyzed using the Mann–Whitney *U* tests (*p* > 0.05). Results: The SEM analysis showed a smaller microgap at the implant–abutment interface in the control group (0.270 μm) than in the test group (3.902 μm). Statistically significant differences were observed between both groups (*p* < 0.05). Conclusions: The use or not of transepithelial abutments affects the microgap size. The transepithelial abutments group presented lower microgap values at the interface with the implant than the Ti-base group in monolithic zirconia partial implant-supported fixed prostheses. However, both groups had microgap values within the clinically acceptable range.

## 1. Introduction

Currently, dental implantology is a highly predictable treatment, with high success rates, being the first choice of treatment in many cases [1,2]. There are multiple restorative options for implants. Depending on the type of retention, it can be screw-retained or cemented [3,4]. It can be restored directly to the implant or using transepithelial abutments, which can be of different heights [5], macrogeometries [6,7,8], and materials [9,10]; they also can be original or compatible abutments [11]. Abutment height has been reported to influence peri-implant marginal bone loss, with several articles showing a reduction in bone loss with increased abutment height [5,12]. On the other hand, it has been shown that the macroscopic design, surface topography, and manipulation of the implant abutment do not influence peri-implant bleeding on probing (BOP). On the contrary, differences in BOP have been reported with several materials, with higher BOP values in titanium compared to zirconia abutments [8]. 

Concerning the materials, nowadays, zirconia is one of the most frequently used. It can be manufactured via CAD-CAM technology (Computer-Aided Design and Computer-Aided Manufacturing) and is usually cemented to a Ti-base [13,14]. 

Two-piece implants have a critical area: the interface or junction between the implant and the abutment. A microgap may appear in this area, which is the distance between the contact surface between the dental implant and the abutment or prosthetic component [13,14,15]. This microgap can be a source of micromovements [16,17] and bacterial contamination [16,17,18,19], where bacteria flow freely between the oral environment and the internal cavity of the implant. This passage of bacteria in addition to toxic bacterial by-products can produce a persistent inflammatory process of the peri-implant tissues, causing peri-implant mucositis, peri-implantitis, and, finally, resorption in the bone crest. In implants with peri-implantitis, the most common bacterial species are *Porphyromonas gingivalis*, *Treponema denticola*, and *Tannerella forsythia* [20]. Factors such as the type of connection, the use of abutment, as well as the torque influence the stability of the implant–abutment connection, which may favor bacterial contamination and the clinical consequences that this entails [21,22,23]. Therefore, a stable connection between the implant and the abutment is going to be sought to reduce these micromotions and the possible consequences of these [16,17]. This connection seems to be the internal conical connection, with multiple authors reporting greater stability of the conical connection compared to the rest [22,24,25].

As a consequence of the misfit between the implant and the abutment, mechanical complications can also occur, such as stress on the prosthetic screw, causing loosening and fracture of the screw; implant overloading; and abutment rotation or fracture [26]. 

The connection of the prosthetic restorations through a transmucosal component, the abutment, enables the transmission of functional masticatory forces. In addition, through the biological seal, where the soft tissues adhere to the abutment surface, the implants are protected from the contaminated oral environment. Consequently, the peri-implant hard tissues are protected from bone resorption [27]. 

Nowadays, the first treatment option for partial or total edentulism is dental implants and fixed dental prostheses, except contraindications. Whether or not to use a transepithelial abutment is one of the issues to be considered when rehabilitating implants; however, few studies compare both treatment options. Therefore, the aim of this in vitro study was to evaluate the microgap at the implant–abutment interface (IAI), analyzed using a scanning electron microscope in three-unit monolithic zirconia implant-supported fixed dental prostheses on transepithelial versus Ti-base abutments. The null hypothesis was that there were no statistically significant differences in the microgap between monolithic zirconia partial implant-supported fixed prostheses connected to transepithelial abutments and connected to Ti-base abutments.

## 2. Materials and Methods

### 2.1. Study Design and Implant–Abutment System Characteristics

A total of 60 commercially titanium dental implants with a conical connection and 3.5 mm diameter and 11.5 mm length (Avinent Ocean, Avinent Implant System, Barcelona, Spain) were divided into two groups (*n* = 30) according to the type of abutment-making three-unit bridges made of monolithic zirconia milled from blocks (DDcubeOne^®^ Multilayer, Dental Direkt GmbH, Herford Germany) and designed using CAD-CAM (Exocad Rijeka 3.1, Exocad GmbH, Darmstadt, Germany).

**Figure 1:**
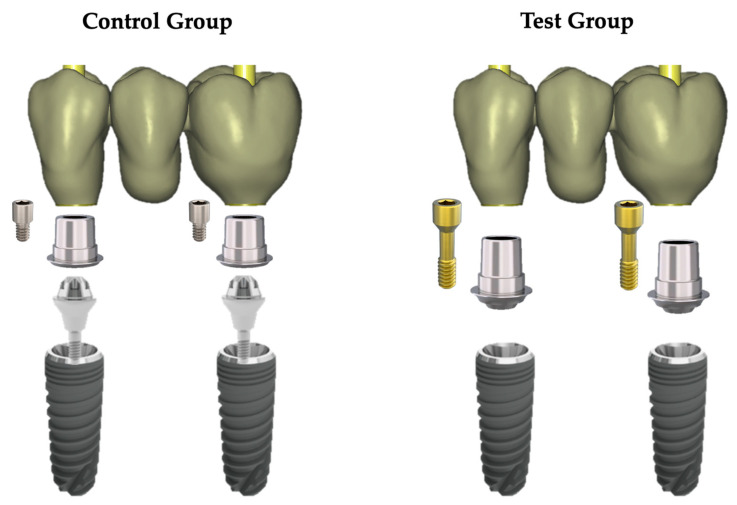
Prosthetic components of the specimens of both groups.

In the control group, 2 mm high transepithelial abutments (Reference 3119; Avinent Ocean, Avinent Implant System, Barcelona, Spain) were screwed to the implants. Three-unit monolithic zirconia bridges cemented to rotatory Ti-bases (Reference 2822; Avinent Ocean, Avinent Implant System, Barcelona, Spain) were screwed. In the test group, there were no transepithelial abutments. The 3-unit monolithic zirconia bridges were cemented to rotary Ti bases (Reference 3050; Avinent Ocean, Avinent Implant System, Barcelona, Spain) and screwed directly to the implants. The prosthetic components of both groups are shown in Figure 1.

### 2.2. Specimen Preparation

Thirty standardized machined polymethylmethacrylate resin blocks (45 mm long, 15 mm wide, and 18 mm high) with an elastic modulus of 3000 MPa (approximate cancellous bone modulus of 1507 MPa) were fabricated with two perforations (12 mm long and 5.5 mm wide) to store the implants.

First, two implants were embedded in each methacrylate block with autopolymerizing acrylic resin (Probase Cold, Ivoclar Vivadent^®^, Schaan, Liechtenstein). The implants were placed at the “supracrestal” level of the methacrylate block to allow the microgap to be observed and measured. In the control group, the two transepithelial abutments were screwed to the implants, and the scan bodies (Reference 2800; Avinent Implant System, Barcelona, Spain) were placed on them. The position of the implants was digitized using a laboratory scanner (Medit T500, Medit Corp., Yeongdeungpo-gu, South Korea). The obtained STL file was employed to design the three-piece monolithic zirconia bridges (anatomy of 1st premolar, 2nd premolar (pontic), and 1st molar) by using CAD program software (Exocad Rijeka 3.1, Exocad GmbH, Darmstadt, Germany). Once a dental technician completed and validated the design, it was sent to the milling center (Avinent CAD CAM, Barcelona, Spain) to obtain 60 zirconia bridges (30 per group). This procedure was repeated in the test group, in which no transepithelial abutments were employed. In this group, the scan bodies (Reference 3080; Avinent Implant System, Barcelona, Spain) were directly screwed to the implants to digitize the implant positions. 

The 3-unit restorations were milled from 14 mm thick zirconia discs (4Y-TZP) (DDcubeOne^®^ Multilayer, Dental Direkt GmbH, Herford, Germany) using a 5-axis precision milling machine (Roland DWX-52DC; Roland DG Deutschland GmbH, Viersen, Germany). According to the properties reported by the company, this material has 1200 ± 150 MPa strength and excellent esthetic properties. Subsequently, to complete the fabrication process, the bridges were placed in a sintering furnace (Ivoclar Vivadent Programat S1, Ivoclar Vivadent^®^, Schaan, Liechtenstein). Then, the bridges of both groups were cemented at the different Ti bases using a permanent self-curing composite cement (Multilink hybrid abutment, Ivoclar Vivadent^®^, Schaan, Liechtenstein). 

With the bridges screwed on the model specimens of each group placed inside a box, a transparent silicone print (Exaclear, GC Ibérica Dental Products, S.L.) was taken to position all the bridge–implant complexes in the same position on each polymethacrylate block. Each bridge–implant complex was placed with the bridge part in the silicone impression, and acrylic resin was poured into the methacrylate blocks until the implants were completely covered. After the polymerization of the acrylic resin, the silicone print was removed, leaving the complex bridge–implants joined in the methacrylate blocks.

The transepithelial abutments and the direct-to-implant Ti-base abutment screws were torqued at 35 Ncm according to the manufacturer’s instructions. An original brand-new wrench was used (Avinent Implant System, Barcelona, Spain). In the control group, the screw connecting the bridge with the transepithelial abutment was tightened at a torque of 20 Ncm according to the manufacturer’s recommendations. Subsequently, screw cavities were filled with a 0.2 mm thick polytetrafluoroethylene (PTFE) tape (PTFE, polytetrafluoroethylene; MIARCO) [28] and definitive restorative material (Tetric Evoceram, Ivoclar Vivadent^®^, Schaan, Liechtenstein). 

### 2.3. Aging Processes

To simulate artificial deterioration, all specimens were subjected to a thermocycling process of 10,000 cycles in distilled water at a minimum temperature of 5 °C and a maximum temperature of 55 °C. The total time for each cycle was 50 s:20 s in the container of cold water (5 °C), 10 s of transition, and 20 s in the container with hot water (55 °C) (VA55, EuroOrtodoncia, Madrid, Spain) [29].

**Figure 2:**
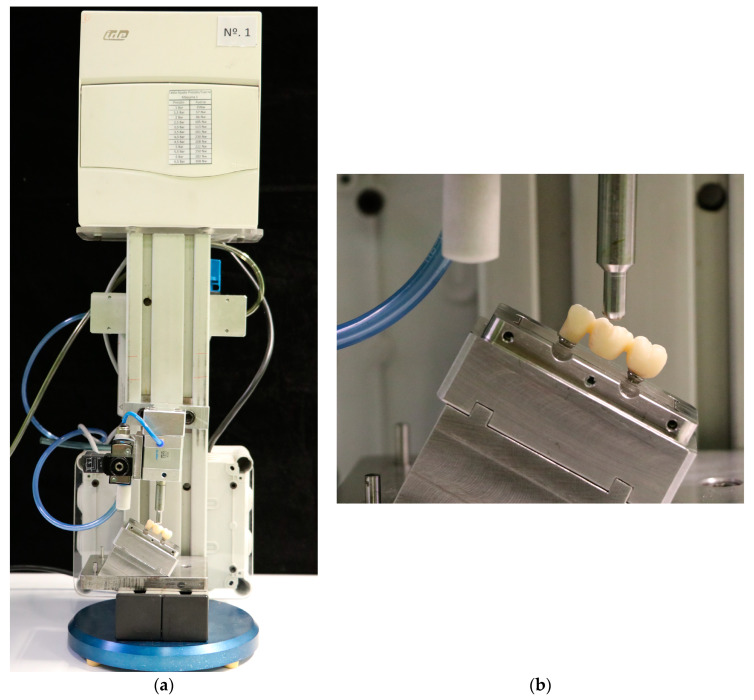
Chewing simulation machine. (**a**) Specimen placed in metal box with 30 degrees of inclination; (**b**) representative image of load application.

After thermocycling, each specimen was subjected to cyclic loading in a chewing simulation machine (Instron^®^, Euroortodoncia S.A. Zwick/Roell testXpert ll Software) (Figure 2a). Each specimen was placed in a metal box with a 30° inclination, where it was firmly secured to avoid displacements during the process (Figure 2b). The load was applied using a deformation-resistant metal element with a hemispherical counting surface to transfer the load on the occlusal surface of the pontic of the 3-unit monolithic zirconia bridge for 300,000 cycles under loads of 200 N at 2 Hz [30].

Followed by thermocycling and chewing simulation, all specimens were inspected under an optical microscope (Telecentric Objective 1:1, Jos. Schneider Optische Werke GmbH, Rhineland-Palatinate, Germany) at 4 magnification Toupview V.x643.7.6701 (Toupview, Toup Tek, ToupTek Photonics Co., Ltd., Hangzhou, China) to verify that there were no alterations or fractures in the structures. In addition, a tactile motion test was performed with dental pliers. 

Before the assessment with the SEM, all the samples were cleaned with steam at a working temperature of 135 °C, with a maximum pressure of 4 bars (VK300, Ivoclar Vivadent^®^, Schaan, Liechtenstein).

### 2.4. Scanning Electron Microscope (SEM) Test

An implant–abutment interface microgap is defined as the microscopic space between an implant and abutment [13,17]; that is, the lack of contact measured from the implant platform to the corresponding contact area of the abutment (Figure 1). 

**Figure 3:**
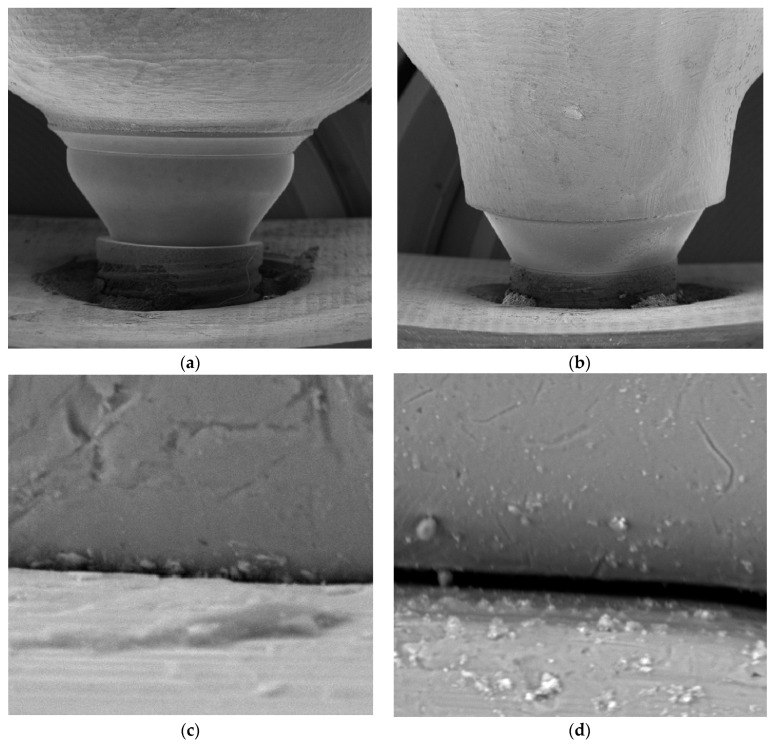
Representative SEM images for microgap assessment. (**a**) Control group specimen (10 magnifications); (**b**) test group specimen (10 magnification); (**c**) control group specimen (1000 magnifications); (**d**) test group specimen (1000 magnification).

The IAI microgap of all specimens was evaluated by using an SEM (JSM-6400, JEOL, Tokyo, Japan). All samples were coated with 24 Kt, 19.32 g/m^3^ density gold using the Q15RS metallizer (Quorum Technologies, Sussex, UK). Subsequently, they were placed perpendicular to the SEM optical axis (90° ± 25°), and the distance measurement was performed parallel to the axis of the implant–abutment interface. Then, the IAI microgap was evaluated in 6 areas (mesiobuccal, buccal, distobuccal, mesiolingual, lingual, and distolingual). These points were previously marked with a permanent marker. Images were obtained from each point at 10, 500, and 1000 magnifications for further evaluation (Figure 3). 

**Figure 4:**
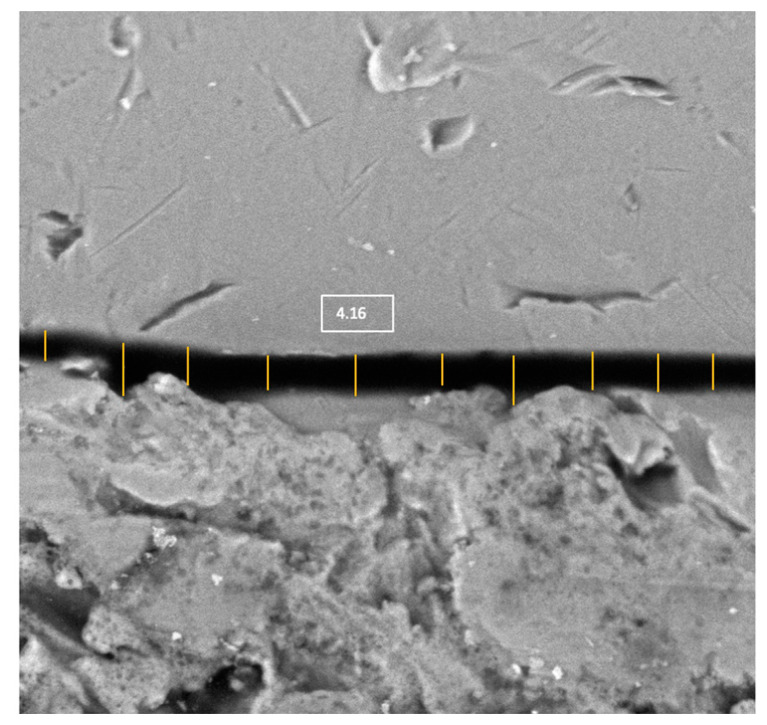
SEM image (1000 magnification) measurements corresponding to the buccal point of the molar of specimen 1 of the Ti-base group.

Microscopic observation and imaging were performed by a microscopy specialist independent of the study (AM.V.M.) (Spanish National Centre for Electron Microscopy ICTS; Complutense University of Madrid, Madrid, Spain). A previously trained operator (R.C.) performed 10 measurements in each of the images, corresponding to the 6 evaluated areas of each IAI, using a computer program (ImageJ64, National Institutes of Health, version J64, Bethesda, MA, USA), thus obtaining 60 images for each IAI (Figure 4). Previous to the measurements, the scale was calibrated. The microgap values obtained were in microns (μm).

### 2.5. Statistical Analysis 

Data were analyzed using a statistical software program (IBM SPSS Statistics for Windows, v.26.0; IBM Corp., Armonk, NY, USA). For the descriptive analysis, the mean, median, and standard deviation values per group were analyzed. The normality of the data distribution was evaluated using the Shapiro–Wilk test (*p* < 0.05). The data were not normally distributed. Therefore, the microgap was analyzed using the Mann–Whitney U test between the transepithelial abutment and Ti-base abutment groups. The confidence level was 95%, so *p*-values less than 0.05 were considered statistically significant.

## 3. Results

**Figure 5:**
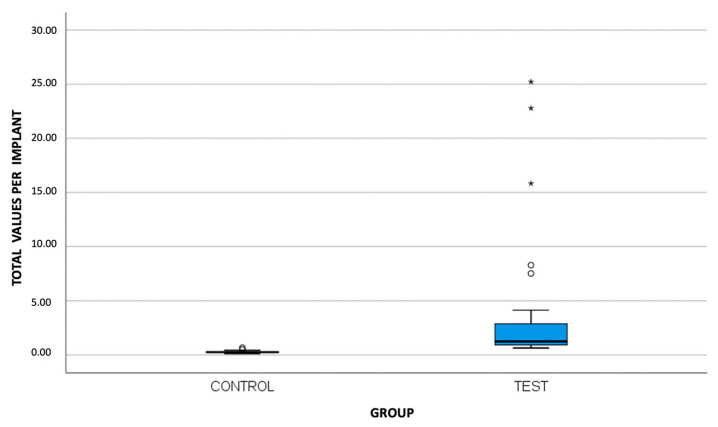
Representative box plot for microgap (μm) at the implant–abutment interface according to total values per implant.

**Figure 6:**
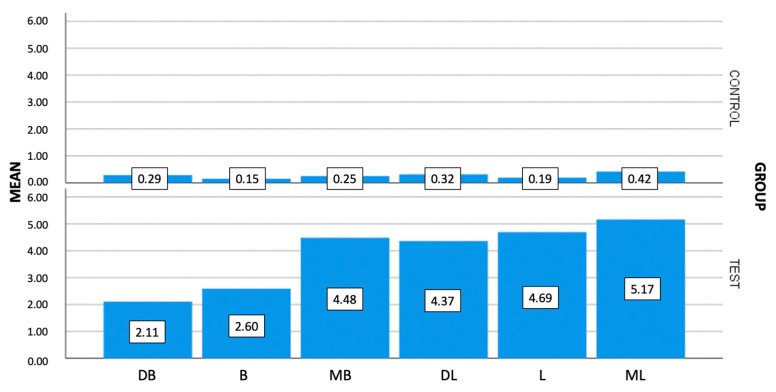
Representative bar chart of mean misfit values (μm) among the evaluated groups per area (MB: mesiobuccal, B: buccal, DB: distobuccal, ML: mesiolingual, L: lingual, DL: distolingual).

No complications occurred in the specimens after thermocycling, chewing simulation, and SEM microgap evaluation, so data from all samples were included for statistical analysis. Six measurements per implant were performed, obtaining 180 per group, with a total of 360 data points. Descriptive statistics were presented for each of the evaluated areas (mesiobuccal, buccal, distobuccal, mesiolingual, lingual, and distolingual) and per implant (mean of all points) (Figure 5 and Figure 6; Table 1). 

The Mann–Whitney U test showed significant differences between the transepithelial abutment and Ti-base groups (*p* < 0.05) in each area and the total number of them per implant. (Table 2). 

## 4. Discussion

This in vitro study aimed to evaluate the presence of microgaps analyzed using a scanning electron microscope in monolithic zirconia partial implant-supported fixed prostheses on transepithelial abutments versus Ti-base abutments. Significant differences in the microgaps of both groups were found, with the transepithelial abutment group presenting a smaller microgap, so the null hypothesis was rejected. 

The misfit between the implant and the abutment or prosthetic component will be critical in clinical situations due to the possible mechanical and biological complications that it may promote [21,22,23,26,27], so its evaluation must be based on reliable, accurate, and reproducible methods. Several methods have been proposed to assess microgaps. Among the different techniques are the use of optical and scanning electron microscopes, as well as radiographic evaluation in two and three dimensions [13,31,32,33,34,35,36,37,38,39]. However, there is currently no consensus on the superiority of the different assessment methods [40]. The present study was based on the evaluation of microgaps via SEM analysis. Its use has been widely documented as a noninvasive, reproducible, and reliable method [32] for assessing microgaps or misfits of prosthetic components. However, SEM analysis has limitations since only two-dimensional images can be obtained. Other imaging techniques, such as digital tomography or microtomography, have been proposed to overcome these limitations. Nevertheless, they are more expensive and require specific equipment and more time for processing and evaluating the images obtained [35,36,37,38,39]. 

The microgap between implant and abutment in conical connection implants should be evaluated in the axis or plane of the contact surface between the implant and abutment. In this regard, the appropriate angle in assessing the microgap should be 90° with respect to the vertical axis of the implant. Due to the proposed prosthetic configuration, the SEM had to be angled at 90° with an angulation variation of ±25° toward the coronal axis to obtain an adequate axis for microgap evaluation. Therefore, the results obtained should be carefully evaluated considering this angulation setting to allow the observation of the microgap [13].

In recent years, there has been a significant increase in the designs and different components and materials of implants and prosthetic components, accompanied by the evolution of CAD-CAM technology. However, two-piece implants still inevitably present a microgap at the interface between the implant and the abutment or prosthetic component [41,42]. This microgap may favor the appearance of possible biological and mechanical complications [12,21,22,23,27]. Therefore, microgap size factors should be considered to achieve an optimal fit between implants and abutments.

Although numerous studies evaluate the existing microgap at the implant–abutment interface and show which microgap values are acceptable, there is no consensus in the scientific literature on the clinically accepted microgap value [43]. Thus, the clinically accepted misfit range is from 10 μm to 150 μm [13,21,32,43,44]. Both groups were considered clinically acceptable in the present study as they presented microgap values below this range. The mean sizes of the control and the test groups were less than 10 μm, 0.27 μm, and 3.90 μm, respectively. The largest microgap size was found in the test group (49.50 μm), and the minimum value was found in both groups (0.11 μm). Consistent with these results, similar results are found for tapered connection implants in abutments fabricated in zirconia (2.57 μm) [13] (2.7–4 μm) [45], milled titanium (0 μm) [31] (1.8–5.3 μm) [45], and in milled cobalt chrome (2.26 μm) [13]. However, the microgap size increases in laser sintering techniques for Co-Cr abutments (13.60 μm), as well as for cast structures (18.40 μm) [13]. In an in vitro study evaluating the fit of three-unit implant-supported, screw-retained frameworks on three implants, it was shown that the CAD/ CAM-fabricated zirconia and CAD/CAM-fabricated cobalt–chromium frameworks were associated with lower misfit values. The misfit values were 5.9 ± 3.6 mm for CAD/ CAM-fabricated zirconia, 1.2 ± 2.2 mm for CAD/CAM-fabricated cobalt–chromium frameworks, 11.8 ± 9.8 mm for conventionally fabricated cobalt–chromium frameworks with pre-machined abutments, and 12.9 ± 11.0 mm for the conventionally manufactured frameworks with castable abutments [45]. Therefore, the manufacturing method affects the accuracy of single crowns and three-unit FDP frameworks. However, the available scientific research is limited regarding the use of transepithelial abutments vs. Ti-base or direct-to-implant frameworks for multiple restorations.

The aging phase is intended to simulate, at a preclinical level, the intraoral conditions to which the restorations would be subjected clinically. The present study used artificial mastication via cyclic loading and thermocycling tests. Thermocycling is based on subjecting different prosthetic components or materials to temperature changes for a certain number of cycles. Similarly, using artificial saliva and distilled water has been described as an immersion medium. In the present study, distilled water was used at a standard temperature of 5 and 55 °C for 50 s each cycle, subjecting the specimens to 10,000 cycles. Considering the ISO standard for this methodology, using both a dry test and physiological saline for the artificial aging of implant restorations is accepted [30]. In the present study, dry static loading was used due to the characteristics of the chewing simulation machine used. The determination of the loading cycles was based on previous studies which reported that a loading process of between 240,000 and 250,000 cycles in a chewing simulation machine corresponds to approximately one year of clinical chewing function in the oral cavity [46], with 300,000 cycles under 200 N loads applied for each specimen. Compared with the previous literature, the parameters used were within current standards in similar studies, ranging from a minimum of 1000 to 1,200,000 cycles for crowns and 10,000 to 2,000,000 cycles for three-unit fixed dental prostheses (FDPs). The applied force varied between 20 and 300 N for crowns and 49 and 200 N for three-unit FDPs [47].

Some limitations of this study should be considered. First, the oral environment, dynamic occlusion, and biological parameters could not be simulated. Therefore, the data obtained cannot always be extrapolated clinically. The microgap was evaluated with an SEM, as in previous studies. Another limitation that could be contemplated is that the present study only assessed the conical connection and two types of abutments, with there being multiple designs of connections, abutments, and restorations depending on the material and type of fabrication. In addition, the microgaps before aging were not recorded, so it is impossible to distinguish if the observed differences were due to the initial device or the aging procedure.

Therefore, future clinical investigations are needed, including different types of connections, different abutment designs, materials, and manufacturing techniques. 

## 5. Conclusions

Within the limitations of this in vitro study, it can be concluded that the use of transmucosal abutments can provide lower implant–abutment-interface microgaps when compared to direct titanium-base abutments for monolithic zirconia partial implant-supported fixed prostheses. Both groups presented microgap values within the clinically acceptable range.

## Figures and Tables

**Table 1:** Descriptive statistics for microgap (μm) per assessed area and group.

Group	Area	Mean	Standard Deviation	Median	Minimum	Maximum
Control group (transmucosal abutment) (N = 30)	MB *	0.25	0.17	0.22	0.11	0.78
	B *	0.15	0.10	0.11	0.11	0.45
	DB *	0.29	0.28	0.23	0.11	1.52
	ML *	0.42	0.31	0.34	0.11	1.20
	L *	0.19	0.19	0.11	0.11	0.99
	DL *	0.32	0.18	0.29	0.11	0.74
	T *	0.27	0.12	0.25	0.11	0.65
Test Group (Ti-base) (N = 30)	MB *	4.48	8.70	0.77	0.11	37.08
	B *	2.60	4.17	0.94	0.11	17.77
	DB *	2.11	2.36	1.12	0.11	10.41
	ML *	5.17	11.24	0.98	0.33	49.50
	L *	4.69	9.18	1.11	0.11	39.91
	DL *	4.37	9.09	1.26	0.11	46.92
	T *	3.90	6.30	1.25	0.64	25.22

* MB: mesiobuccal, B: buccal, DB: distobuccal, ML: mesiolingual, L: lingual, DL: distolingual, T: total per implant.

**Table 2:** Comparison of microgap (μm) measured between transmucosal abutment and Ti-base abutment groups using Mann–Whitney U test.

	Area						
	MB Gap	B Gap	DB Gap	ML Gap	L Gap	DL Gap	T Gap
Mann–Whitney U	54.50	26.50	56.50	109.00	37.00	67.00	1.00
Z	−5.87	−6.47	−5.84	−5.05	−6.24	−5.67	−6.64
Significance *	0.001	0.001	0.001	0.001	0.001	0.001	0.001

MB: mesiobuccal, B: buccal, DB: distobuccal, ML: mesiolingual, L: lingual, DL: distolingual, T: total per implant. * Significance level at 0.05.

## Data Availability

The data presented in this study are available on request from the corresponding author.
